# The Intentional Stance Test-2: How to Measure the Tendency to Adopt Intentional Stance Towards Robots

**DOI:** 10.3389/frobt.2021.666586

**Published:** 2021-10-07

**Authors:** Nicolas Spatola, Serena Marchesi, Agnieszka Wykowska

**Affiliations:** Social Cognition in Human-Robot Interaction Laboratory, Italian Institute of Technology, Genova, Italy

**Keywords:** human-robot interaction (HRI), intentional stance theory, mentalization, measurement, social robotics/HRI

## Abstract

In human-robot interactions, people tend to attribute to robots mental states such as intentions or desires, in order to make sense of their behaviour. This cognitive strategy is termed “intentional stance”. Adopting the intentional stance influences how one will consider, engage and behave towards robots. However, people differ in their likelihood to adopt intentional stance towards robots. Therefore, it seems crucial to assess these interindividual differences. In two studies we developed and validated the structure of a task aiming at evaluating to what extent people adopt intentional stance towards robot actions, the Intentional Stance task (IST). The Intentional Stance Task consists in a task that probes participants’ stance by requiring them to choose the plausibility of a description (mentalistic vs. mechanistic) of behaviour of a robot depicted in a scenario composed of three photographs. Results showed a reliable psychometric structure of the IST. This paper therefore concludes with the proposal of using the IST as a proxy for assessing the degree of adoption of the intentional stance towards robots.

## Introduction

### Intentional Stance Towards Robots

Humans readily attribute intentionality and mental states to living and non-living entities such as robots (Fisher, 1991; Fletcher et al., 1995; Epley et al., 2007). Dennett (Dennett, 1971; Dennett, 1988) suggested that individuals may use different strategies when trying to predict the behaviour of various entities or systems. The “physical stance” works with intuitive notions of physics and it is used whenever a person tries to predict, for instance, the trajectory of a falling object. However, the actions of agents who produce more complex patterns (such as humans) cannot be predicted by applying these rules alone. A more efficient strategy (in terms of predictions) would be to adopt the “intentional stance,” which assumes that mental states are the underlying explanations of the observed behaviour (Thellman et al., 2017; De Graaf and Malle, 2019).

Within this context, social robots represent a particular category of artefacts explicitly designed to potentially elicit the adoption of the intentional stance (for a review see Perez-Osorio and Wykowska (2020). Literature suggests that adopting the intentional stance in Human-Robot Interaction (HRI) may bias how humans behave towards robots. For instance, the attribution of mental states to robots increases the acceptance of robots with more positive attitudes (Eyssel et al., 2012), anthropomorphic attributions (Spatola et al., 2019; Spatola and Wudarczyk, 2020), trust toward them (Waytz et al., 2014), or the likelihood to engage in pro-social behaviours (Riek et al., 2008; Spatola, 2019b).

The present study aimed at further development and validation of the Intentional Stance test (Marchesi et al., 2019) which measures the degree of adopted intentional stance towards robots. We aimed at delineating (exploratory factor analysis) and confirming (confirmatory factor analysis) the tool’s factor structure, and offering three tests of convergent validity using different, but theoretically related, constructs (i.e., anthropomorphism, attitudes toward robots and personality traits).

### Intentional Stance and Correlated Constructs Relative to Human-Robot Interaction

Because adoption of the intentional stance (in HRI) is related to perception of robots as intentional agents, it may also interplay with other core phenomena in HRI such as anthropomorphism (i.e. the attribution of human characteristics to non-humans) (Epley et al., 2007; Waytz et al., 2010b) and attitudes toward robots (i.e. the “state of mind” of an individual or a group toward an object, an action, another individual or group) (Evans, 2008; Albarracin and Shavitt, 2018; Spatola and Wudarczyk, 2020). We develop the theoretical link between intentional stance and anthropomorphism, as well as general attitudes towards robots as a basis for convergent validity tests for our tool measuring the tendency to adopt the intentional stance.

#### Intentional Stance and Anthropomorphism

The Intentional stance may be considered as a concept related to anthropomorphism albeit different. Anthropomorphism is a broader concept which denotes attribution of human characteristics to non-humans, ranging from physical attributes (seeing “faces” in the shapes of clouds) to mental attributes. Intentional stance, on the other hand, refers solely to the inference of mental states. Although different, intentional stance and anthropomorphism remain correlated concepts sharing communalities (Waytz et al., 2010b). Therefore one would expect that in the case of HRI, the higher degree of adoption of intentional stance, the higher the degree of anthropomorphising a robot.

#### Intentional Stance and Attitudes Toward Robots

The Intentional stance also depends on the general set of attitudes one may have toward robots. Attitudes towards robots (as toward humans) are reliable predictors of the type of inferences and behaviours (e.g. positive/negative) one generates with respect to a given subject (Ajzen and Fishbein, 1977; Maio and Haddock, 2009). For instance, negative attitudes toward a social group tend to result in the deprivation of human characteristics, i.e., dehumanization. People belonging to this group would be considered with low level of agency or rationality. On the other hand, they would be imbued with mechanical characteristics such as passivity or inertness (Haslam, 2006).

### Intentional Stance and Personality

Interindividual variability related to the tendency to adopt the intentional stance towards robots may be explained with reference to personality traits (Ghiglino et al., 2020). For instance, the need for cognition (i.e. the extent to which individuals are inclined towards effortful cognitive tasks) or need for closure (i.e. the need to alleviate ambiguity) have been theorized as two central predictors of adoption of intentional stance when observing non-human agents’ actions (Roets and Van Hiel, 2011; Spatola and Wykowska, 2021).

#### The Need for Cognition

The Intentional stance entails inductive inference from available information to interpretation of behaviour in terms of goal-directed actions (Barsalou, 1983; Epley et al., 2007; Urquiza-Haas and Kotrschal, 2015; Spatola, 2019a). Inferences do not rely only on purely external phenomena but also on the representation of the observed agent available to the observer at the time of judgment (Noce and Utter, 1975). Hence, attributions of mental states might be a result of an interplay between the natural tendency to use internal knowledge about humans to represent non-humans (Baker et al., 2009), and a process of controlling this default tendency by means of using more accurate representation and information (Evans, 2008; Urquiza-Haas and Kotrschal, 2015). The individual tendency towards engaging in this cognitively demanding control process is called the need for cognition (Cacioppo and Petty, 1982). Cacioppo and Petty defined the need for cognition as the inner pleasure created by an individual’s effort to process complex information (Cacioppo and Petty, 1982). Therefore, the more people are likely to engage in effortful processing, the less tendency towards adopting the intentional stance they should exhibit. This entails that individuals who have a high degree of need for cognition should be more open for using alternative and more accurate (mechanistic) representation of robot actions (Spatola and Wykowska, 2021).

#### The Need for Closure

The Intentional stance also provides an intuitive and readily accessible strategy for reducing the contextual complexity and uncertainty of an environment (Dennett, 1971; Epley et al., 2007) by providing familiar explanations (reference to mental states) of unknown complex phenomena (behavior of unfamiliar complex agents such as robots). Adopting the intentional stance should, therefore, be influenced by the motivation to resolve uncertainty, seek meaning, and feel efficacious. The Need for Closure concept was introduced to develop a theoretical framework for inter-individual differences in this cognitive-motivational aspect of decision making (Kruglanski, 1990; Kruglanski and Webster, 1996). Webster and Kruglanski (Webster and Kruglanski, 1994) proposed a five-dimension taxonomy of the need for closure including 1) the *need for order*, the preference for structure and avoidance of disorder. 2) The *need for predictability*, as the aptitude for secure and stable knowledge. 3) The *need for decisiveness*, as the search for clear decision making*.* 4) the *discomfort toward ambiguity,* as the negative experience in situations devoid of clarity. And finally, 5) the *close-mindedness*, as the unwillingness to challenge one own knowledge by alternative opinions or inconsistent evidence. People with a high need for closure tend to ground their reasoning on more accessible information and familiar way of reasoning about others’ behaviour (reference to mental states) rather than build an effortful, but (perhaps) more accurate representation (mechanistic in the case of robots) l (Spatola and Wykowska, 2021).

### The Present Study

**FIGURE 1:**
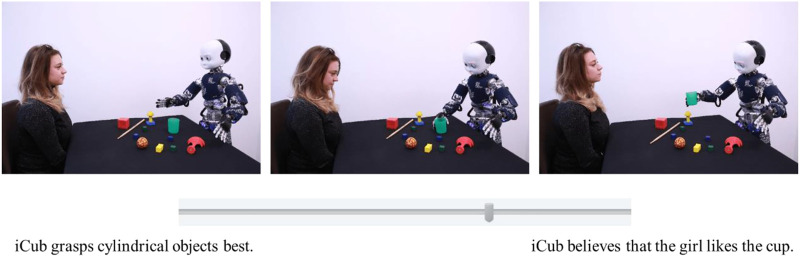
Example of an item from Marchesi et al. (2019).

One of the grand challenges of social robotics is to better understand and evaluate interpersonal differences in attitudes towards robots (Yang et al., 2018). In this study, we examined how individual differences in the need for cognition and the need of closure predict the tendency to adopt the intentional stance, as measured by the Intentional Stance test (IST, Marchesi et al., 2019). In addition, the study aimed at validating the IST. In the first experiment, we tested the psychometric structure of IST, based on the set of raw stimuli used in Marchesi et al. (2019). The set consists of 34 items, each with a sequence of three photographs representing a robot engaged in daily activities (Figure 1). The task requires participants to decide between two descriptions of the scenarios, one using mentalistic and the other mechanistic vocabulary (Marchesi et al., 2019). Participants should choose which description, according to them, describes the scenario better.

To test the validity of the IST, we evaluated the external validity of the task in relation to anthropomorphic attributions (measured by the Human-Robot Interaction Evaluation Scale (Spatola et al., 2020). In addition, we used both the need for cognition (Cacioppo et al., 1984) and need for closure (Roets and Van Hiel, 2011) as convergent measures for the psychometric validity of our task at the individual level. By doing so, we may assess that our measure is anchored in the existing frameworks explaining the attribution of mental capacities to non-human agents (Epley et al., 2007).

In the second experiment, we produced a confirmatory factor analysis and tested the correlation between IST and the attitudes towards robots (measured by the Negative Attitude toward Robots scale (Nomura et al., 2006a)); in addition, we tested whether the IST could predict anthropomorphic attributions to other robots.

## Experiment 1: Task Development

The first experiment aimed to set forth the best factorial matrix (using an exploratory factor analysis, EFA) by selecting the items from Marchesi et al. raw material (Marchesi et al., 2019) that proved to be internally consistent to measure tendency to adopt the intentional stance and to identify the potential factors embedded in this factorial matrix.

First, to identify the structure of the task, we used an exploratory factor analysis^1^. Second, we selected an anthropomorphism scale (Spatola et al., 2020) measuring the agency attributed to a robot agent for convergent validity test (see details in method section). We also used the need for cognition and need for closure constructs to evaluate whether (or not) the mentalization tendency measure could be embedded in the general theoretical framework proposed by Epley et al. (2007).

### Method

The participants were 353 French speakers recruited online through Prolific (M_age_ = 22 years, *SD* = 8.66, 93 males, 206 females and 12 non-declared). All participants gave consent to voluntarily participate in the study. The study was approved by the local ethics committee (Comitato Etico Regione Liguria). The collected data were entirely anonymous. The sample size was determined by the recommendation in exploratory factor analyses (EFA). In EFA, based on the number of items (q = 34), Schreiber et al. (2006) recommend 10 observations resulting in a minimum of 340 required participants.

First, participants had to complete the French version of the 34 Marchesi raw items (Marchesi et al., 2019)^2^. Each item was composed of a scenario and two sentences with a bipolar scale and a 100-point slider between the mechanistic and mentalistic sentences^3^ (one of the sentences was positioned on the left, and the other one on the extreme right of the scale) (Figure 1). In each item, participants were explicitly instructed to move the slider on the bipolar scale toward the sentence that they thought was a more plausible description of the story depicted in the scenario. As illustrated in Figure 1, the two descriptions (mentalistic and mechanistic) were placed at the two ends of the scale. The cursor was initially always placed at the centre of the scale (i.e., the null value). For 50% of the items, the mechanistic sentence was presented on the left side of the slider, while the mentalistic was presented on the right side. For the other 50%, the location of mechanistic and mentalistic sentences was reversed. The order of presentation of the items was randomized.

*Anthropomorphism*. Participants also evaluated the iCub robot on the Human-Robot Interaction Evaluation Scale (HRIES) (Spatola et al., 2020) which contains four dimensions of robot evaluation including Sociability (4 items, e.g., Warm, α = 0.90), Agency (4 items, e.g., Self-reliant, α = 0.73), Animacy (4 items, e.g., Alive, α = 0.63), and Disturbing (4 items, e.g., Creepy, α = 0.83). This scale makes it possible to evaluate static, in motion or interactive robots on a broad spectrum of anthropomorphic attributions. For each item, participants rated whether they agreed or disagreed (from 1 to 7) on the attribute related characteristics to the iCub robot (presented on a picture above the scale). (i.e., “For each trait, you will have to evaluate whether, according to you, it corresponds or not to the robot that is presented to you.”). For each trait, a 7-points slider scale was presented from 1 “not at all” to 7 “totally”. The HRIES was chosen because it addresses the attribution of intentional properties in the agency dimension of the scale and, as such, seems appropriate to test the convergent validity of the IST measure with respect to tendency to adopt the intentional stance.

*Need for cognition.* We administered the short-version of the Efficient Assessment of Need for Cognition (Cacioppo et al., 1984) with a positive dimension that assesses the need for cognition (3 items, e.g., I would prefer complex to simple problems, α = 0.81) and a negative dimension that assesses the aversion for cognition (3 items, e.g., Learning new ways to think doesn’t excite me very much, α = 0.78). For each item, participants rated whether they agreed or disagreed with the statement on a scale from 1 to 7).

*Need for closure.* Participants also completed the short version of the Need for Closure (NFC) scale (Roets and Van Hiel, 2011), which is based on the full NFC scale (Webster and Kruglanski, 1994). The scale includes five dimensions with three items in each dimension representing various ways in which NFC is expressed. The five dimensions are: need for order (e.g., I enjoy having a clear and structured mode of life, α = 0.84), need for predictability (e.g., I dislike unpredictable situations, α = 0.78), need for decisiveness (e.g., When I have made a decision, I feel relieved, α = 0.70), discomfort toward ambiguity (e.g., I don’t like uncertain situations, α = 0.68), and close-mindedness (e.g., I do not usually consult many different opinions before forming my own view, α = 0.76). For each item, participants rated whether they agreed or disagreed with the statement on a scale from 1 to 7).

At the end of the experiment, a commentary box was left open to participants

### Results

Two items from the IST were removed from analyses because 23 participants reported that they were confusing (Items 3 and 15, see supplementary materials at osf.io/z2kpc/).

#### Exploratory Factor Analysis

##### Quality of the Sample Size for Factor Analysis

First, we used Bartlett’s sphericity test to ensure inter-item correlation, χ^2^(496) = 3294.36, *p* < 0 001. Inter-item correlations examine the extent to which scores on one item are related to scores on all other items in a scale (Ruff et al., 1976; Williams et al., 2010). Secondly, we conducted a Kaiser-Meyer-Olkin (KMO) test that ensures that once the linear effect of the other items has been controlled, the partial correlations of each pair of items are low, which would confirm the presence of latent factors linking the items to each other (Williams et al., 2010). Its value varies from 0 to 1. This is an index for measuring the quality of sampling for the factor analysis. We obtained a KMO = 0.93, where values between 0.8 and 1 indicate the sampling is adequate (Dziuban and Shirkey, 1974; Cerny and Kaiser, 1977; IBM, 2011).

##### Analysis Method

We chose a common factor model to attribute the variance to latent factors. This method provides more reliable results than component models (e.g. PCA) in the majority of the cases, while the methods would be roughly equivalent in the remaining cases (De Winter ad Dodou, 2016; Gorsuch, 1990; Snook and Gorsuch, 1989; Velicer and Jackson, 1990; Widaman, 1993). Our analysis method started with a maximum likelihood method of extraction with Promax rotation^4^. The Promax rotation is able to deal with the differences between the high and low factor saturation coefficients by raising them to the power κ (here we considered κ = 4, the default value^5^). When the loadings are raised to a κ th power, they are all reduced resulting in a simple structure. As the absolute value of the coefficients decreases, the gap between them increases (Hendrickson and White, 1964; Maxwell and Harman, 1968; Gorsuch, 1990). We analyzed the pattern matrix, which holds the beta weights to reproduce variable scores from factor scores.

##### Selection of Items

To select the items we used an iterative method in order to optimize the information rate among factors (Spatola et al., 2020). The first pattern matrix produced six factors with an eigenvalue higher than 1 (51.04% of explained variance).

For each factor we proceeded as follows: all items loaded at a minimum of 0.30 on a common factor were included in a scale reliability analysis with the other loaded items in order to evaluate the reliability of this set of items. We considered set of items extracted from factors with an alpha lower than 0.7 as non-reliable and dropped the corresponding factor to ensure the psychometric structure stability (Laher, 2010; Spatola et al., 2020). In each reliable set of items (referring to factors) we evaluated the contribution of each item. An item was dropped if its inclusion did not increase the alpha of the set or its exclusion increases the alpha of the set to maximize the Cronbach’s alpha of the factor (Cronbach, 1951; Unwin, 2013).

**FIGURE 2:**
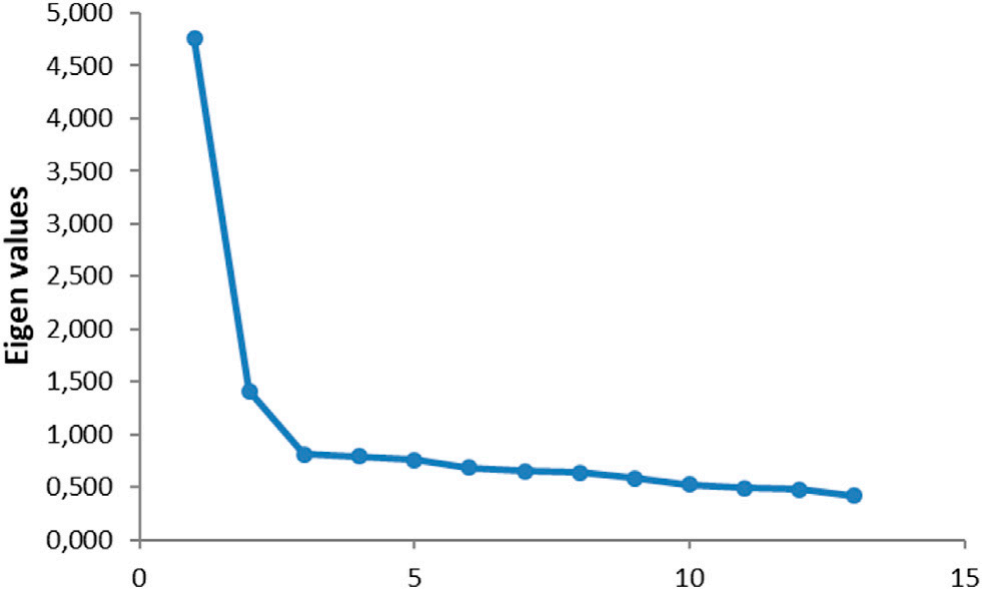
Eigen values for experiment 1 factor analysis.

We conducted a new EFA and applied the same method until the results showed a stable structure. This method offers the advantage to optimize the amount of information extracted by each items. From the 32 experimental items, 13 remained in the final matrix. On this final matrix, one item (i.e. Q12) cross-loaded and was excluded from the factors (Hair et al., 2010). The 12 items remaining loaded on 2 factors, χ^2^(66) = 1006.66, *p* < 0 001; *KMO* = 0.90, explaining 46.78% (compared to the 52.00% with the 7 factors) of variance (Figure 2) with 2 factors (Table 1). In comparison, a single factor structure with the same items resulted in 35.55% of explained variance. The first factor encompasses items in which the robot is interacting with a human while the second factor includes items in which the robot is alone. The two factors were correlated at *r* = 0.55, *p* < 0.001.

**TABLE 1:** Experiment 1 pattern matrix presenting loading factors for each item, percent of explained variance and Cronbach’s alphas for each factor of the final factors. Items in bold are the items included in the final matrix; and factor correlation matrix.

	Factor	
	1	2
Q26	0.78	−0.14
Q25	0.70	0.00
Q21	0.64	−0.16
Q24	0.60	0.08
Q27	0.49	0.24
Q22	0.34	0.19
Q7	−0.05	0.74
Q2	−0.25	0.73
Q31	0.01	0.55
Q14	0.13	0.50
Q17	0.10	0.48
Q34	0.29	0.39
Percentage of explained variance	35.55	11.23
Cronbach’s alpha	0.77	0.74

The mean of the first factor was 50.78 with a SD = 24.21. The mean of the second factor was 30.62 with a SD = 21.53. The min-max of the participants’ responses includes the 100 points of the slider.

**FIGURE 3:**
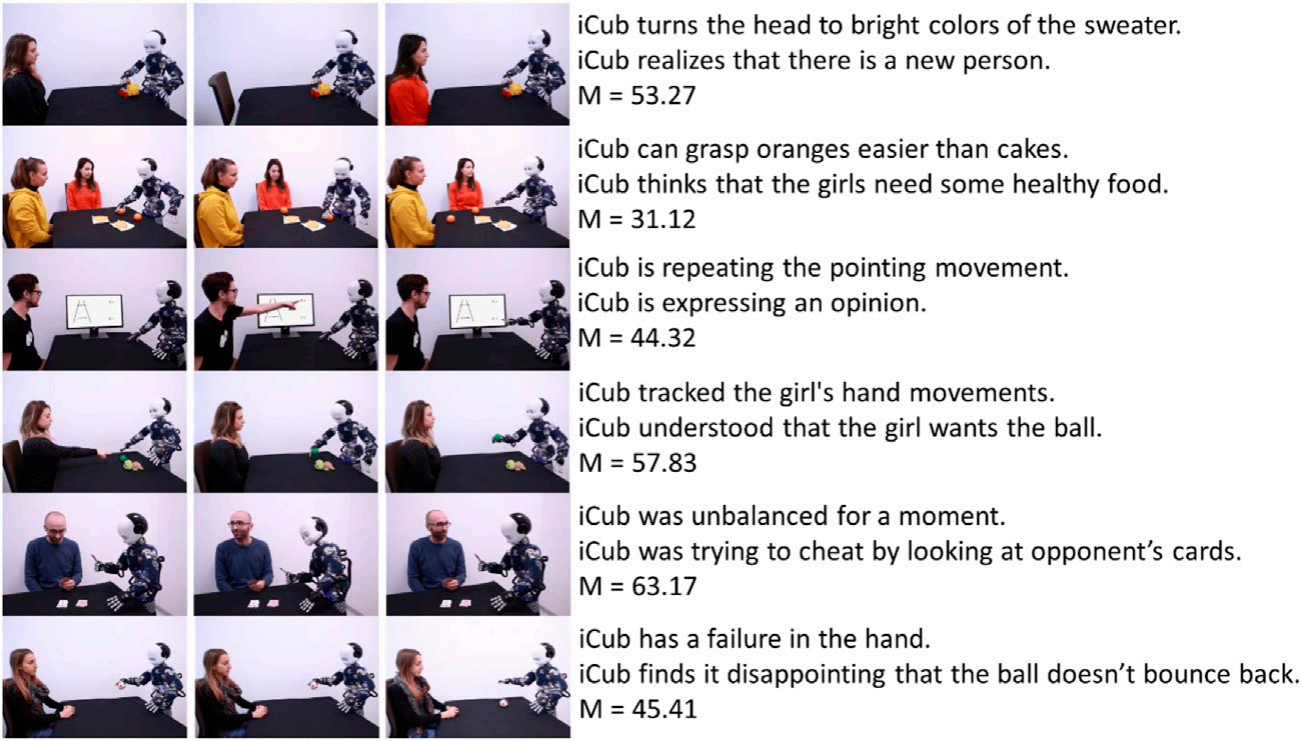
Factor 1 items with mechanistic and mentalistic descriptions, and the average score for each item.

**FIGURE 4:**
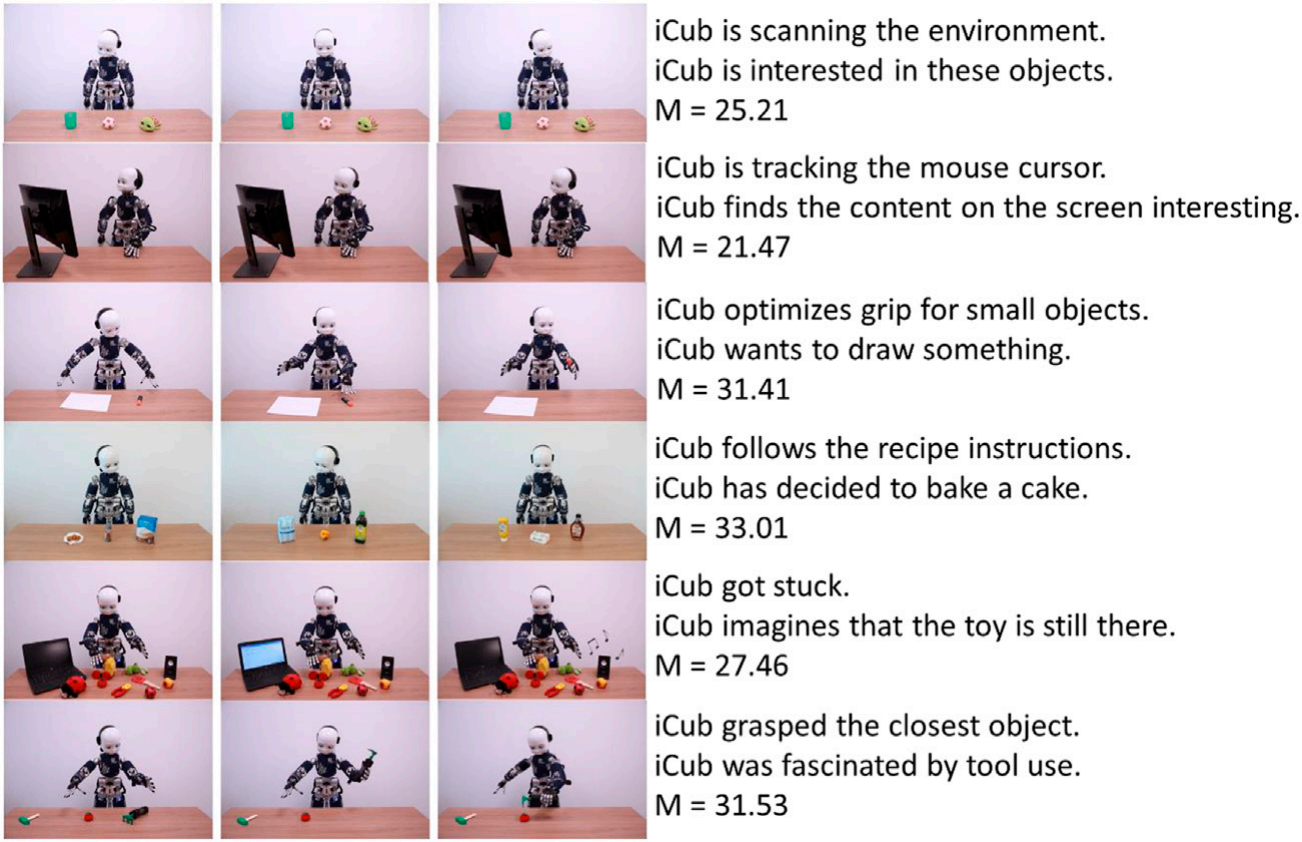
Factor 2 items with mechanistic and mentalistic descriptions, and the average score for each item.

Figure 3 presents the scenarios and their respective mechanistic and mentalistic sentences for Factor 1 and Figure 4 for Factor 2.

#### Tendency to Adopt the Intentional Stance in Relation to Anthropomorphism

To evaluate the consistency of IST’s factors we correlated the IST scores with the HRIES scores (Table 2). The two factors were positively correlated with the dimensions of the HRIES (all *p*_s_ < 0.001) except for the Disturbing dimension (*p*_s_ > 0.100). We also conducted partial correlation analyses controlling for the other factor. Results were similar for agency, sociability and animacy (all *p*_s_ < 0.002) and remain non-significant on disturbing dimension of the HRIES (*p*_s_ > 0.328).

**TABLE 2:** Correlation matrix between the two IST factors and the HRIES dimensions.

		Disturbing	Agency	Sociability	Animacy
Factor 1	Pearson’s r	−0.07	0.39	0.35	0.26
	*p* value	0.173	<0.001	<0.001	<0.001
Factor 2	Pearson’s r	−0.07	0.41	0.44	0.38
	*p* value	0.197	<0.001	<0.001	<0.001

Table 3 presents the correlation matrix for the HRIES dimensions.

**TABLE 3:** Correlation matrix between the four HRIES dimensions.

		Disturbing	Agency	Sociability
Agency	Pearson’s *r*	−0.11	X	X
	*p* value	0.033		
Sociability	Pearson’s *r*	−0.32	0.63	X
	*p* value	<0.001	<0.001	
Animacy	Pearson’s *r*	−0.24	0.43	0.48
	*p* value	<0.001	<0.001	<0.001

#### Intentional Stance in Relation to the Individual Differences in Need for Cognition and Need for Closure

To test the external validity of the two factors, we tested their correlation with need for cognition and need for order (Epley et al., 2007). The results (all *p* < 0.001) supported the hypothesis stated that participants declaring higher level of need for cognition (or low level of aversion for cognition) and high need for closure should demonstrate higher tendency to adopt the intentional stance toward robots (see Table 4).

**TABLE 4:** Correlation matrix the two factors, the HRIES and the need for cognition and need for closure dimensions.

		Aversion for cog.	Need for cog.	Order	Predict.	Decisiveness	Ambigu.	Closemind.
Factor 1	Pearson’s r	−0.19	0.22	0.19	0.17	0.25	0.19	0.20
	*p* value	<0.001	<0.001	0.001	0.001	<0.001	<0.001	<0.001
Factor 2	Pearson’s r	−0.19	0.26	0.18	0.20	0.23	0.23	0.25
	*p* value	0.001	<0.001	0.001	<0.001	<0.001	<0.001	<0.001
Disturbing	Pearson’s r	−0.08	−0.03	−0.05	−0.10	−0.07	−0.05	−0.05
	*p* value	0.160	0.593	0.362	0.072	0.195	0.389	0.324
Agency	Pearson’s r	−0.06	0.45	0.33	0.31	0.35	0.36	0.30
	*p* value	0.275	<0.001	<0.001	<0.001	<0.001	<0.001	<0.001
Sociability	Pearson’s r	−0.05	0.32	0.26	0.28	0.28	0.35	0.24
	*p* value	0.387	<0.001	<0.001	<0.001	<0.001	<0.001	<0.001
Animacy	Pearson’s r	0.06	0.16	0.15	0.16	0.16	0.13	0.22
	*p* value	0.280	0.003	0.006	0.003	0.002	0.012	<0.001

#### Clustering of the Measures

To better understand how the two factors of the IST interplayed, we processed two-step clustering^6^ using the 12 items to delineate “intentional stance tendency” clusters of participants (Bacher et al., 2004). The clustering proposed a solution with a 3 clusters’ matrices with a 1.42 ratio sizes and a fair cluster quality. According to cluster silhouette and cluster comparison, we argue for a low vs. medium vs. high intentional stance tendency. Clusters differed on Factor 1, *F*(2,352) = 301.24, *p* < 0.001, η^2^_p_ = 0.63, Factor 2, *F*(2,352) = 329.62, *p* < 0.001, η^2^_p_ = 0.65, of the IST (Table 5). All contrasts (with Bonferroni correction) were significant (all *p*_s_ < 0.001).

**TABLE 5:** Description of the cluster solution and Cluster comparison on the IST factors.

		Cluster 1		Cluster 2		Cluster 3	
		Mean	Std. Dev.	Mean	Mean	Mean	Std. Dev.
Factor	1	21.02	14.89	53.11	13.92	70.02	15.38
	2	10.30	9.94	23.63	12.10	52.59	14.94

As an exploratory control analysis we conducted a multivariate analysis including the 3 clusters as predictors of the HRIES dimensions. Results showed a significant effect on agency, *F*(2,352) = 33.02, *p* < 0.001, η^2^_p_ = 0.16, sociability, *F*(2,352) = 37.44, *p* < 0.001, η^2^_p_ = 0.18, and animacy, *F*(2,352) = 23.85, *p* < 0.001, η^2^_p_ = 0.12, but not disturbing, *F*(2,352) = 0.55, *p* = 0.576, η^2^_p_ < 0.01. Contrasts (with Bonferroni correction) were all significant (*p*_s_ < 0.002) with lower anthropomorphic attributions from the cluster 1 compared to cluster 2 and cluster 2 compared to cluster 3.

### Discussion Experiment 1

The first experiment aimed at examining the psychometric structure of Marchesi et al. (2019) material and at identifying inter-individual differences in the tendency to adopt the intentional stance towards robots Based on the factorial analysis we were able to extract 12 items grouped in two factors with reliable fit indices. The two factors were correlated without reaching the 0.80 merging threshold, thereby providing evidence for the two factors structure. The first factor included scenarios which displayed the robot interacting with human(s) (social robot factor) while the second factor consisted of scenarios which displayed the robot acting in isolation (isolated robot factor). Results showed higher intentional stance scores for the social robot compared to the isolated robot factor. We discuss these two factors further in the general discussion.

Interestingly, the scores on both factors were positively correlated with external anthropomorphic attributions in a [0.17, 0.32] range arguing for different constructs and, therefore, the usefulness of IST as a measure of adoption of the intentional stance. In line with literature, and as predicted, we also found that tendency to adopt the intentional stance toward robots is intertwined (albeit distinct) with anthropomorphism (Epley et al., 2007; Spatola, 2019a). Importantly, the tendency to adopt the intentional stance was correlated to the “agency” dimension of the HRIES which is specifically related to the attribution of intentional capacities to robots. This provides evidence for the convergent validity.

We also found a significant correlation with the individual differences in need for cognition and need for closure (as proposed by the framework of Epley et al., 2007). In line with previous results on tendency for anthropomorphism (Spatola and Wykowska, 2021), the higher the need for cognition and need for closure, the more likely an individual will adopt intentional stance towards robot’s actions. Epley et al. (2007) proposed that the need for cognition and closure are important individual factors for attribution of mental states to non-human agents. Accordingly, people with a high need for cognition and closure are prone to finding a strategy which maximizes their feelings of control over the environment. With respect to Epley et al. and Dennett, mentalistic attributions (Dennett, 1971; Epley et al., 2007; Marchesi et al., 2019), increases the predictability and understandability of agents’ behaviour by ascribing them a goal, or an intention (Waytz et al., 2010a; Waytz et al., 2010b). As such, the higher the need for cognition, the higher the likelihood to adopt the intentional stance.

Finally, our cluster analysis showed three (low vs. medium vs. high) clusters of participants based on tendency to adopt the intentional stance. This solution accurately predicted the level of anthropomorphic attributions including the agency dimension, important for the convergent validity.

## Experiment 2: Confirmatory Factor Analysis

As a second step, we tested the validity of the task with a confirmatory factor analysis. Confirmatory factor analysis is typically the next step after exploratory factor analysis in construct validation method. It aims to confirm the model under scrutiny (Worthington and Whittaker, 2006; Wood, 2008; Tabri and Elliott, 2012).

We also tested the external validity of the modified IST (IST-2, which is the original IST modified to include only selected items in the present Experiment 1. We chose to test external validity of IST-2 Based on the link between intentional stance and attitudes toward robots. We used the measure of attitudes toward robots as a comparative test for external validity of the scale, in order to have a different reference than the one used in Experiment 1. In line with past studies showing that negative attitudes toward an agent (or a group) decrease the attributions of mental states (Haslam and Loughnan, 2014; Kteily et al., 2016; Spatola, 2019b), thereby increasing the level of dehumanization, we hypothesized a negative correlation between the level of mentalistic attributions and the negative attitudes toward robots. Conversely, we expected a positive correlation between mentalistic attributions and positive attitudes.

Finally, assuming that the IST-2 measures the likelihood to adopt the intentional stance towards robots, we also investigated the extent to which the scores on IST-2 were related to anthropomorphic attributions in general (rather than specific to the robot presented on the scenario). Indeed, we hypothesized that if the IST-2 measures the tendency of participants to adopt the intentional stance, this tendency should correlate with the tendency to anthropomorphize other robots (i.e. Pepper, NAO), different than iCub, which was presented in the scenarios of IST.

### Method

Participants were 135 French speakers recruited through Prolific (M_age_ = 21.72, SD = 5.26, 30 males, 100 females, and 5 other or non-declared). All participants gave written consent to participate voluntarily in the study. The study was approved by the local ethics committee (Comitato Etico Regione Liguria). The collected data were entirely anonymous. The sample size was determined by the recommendation in exploratory factor analyses (EFA). In EFA, based on the number of items (q = 12), Schreiber et al. (2006) recommend 10 observations resulting in a minimum of 120 required participants.

Participants had to evaluate the 12 scenarios of the IST-2 (as extracted from Experiment 1 EFA) (Figure 1). Scenarios were randomly presented with the mentalistic description on the right or on the left of the scenarios. Similarly to Experiment 1, participants were asked to move a slider towards the description that they thought best fit the displayed scenario.

*Attitudes toward robots*. At the end of the experiment, participants completed Nomura, Kanda and Suzuki’s (Nomura et al., 2006b) scale measuring negative attitudes toward robots, hereafter referred to as NARS scale. The NARS scale constitutes of 14 items in three constructs: social/future implications (6 items, e.g., “I feel that if I depend on robots too much, something bad might happen”) (α = 0.70); emotional attitudes (5 items, e.g., “I would feel uneasy if robots really had emotions”) (α = 0.81); and actual interactions (3 items, e.g., “I would feel very nervous just standing in front of a robot”) (α = 0.71). For each dimension, participants rated whether they agreed or disagreed (from 1 to 7).

*Anthropomorphic inferences*. Participants also filled out The Human-Robot Interaction Evaluation Scale (HRIES) (Spatola, Kühnlenz & Cheng, under review) with the four sub-dimensions of Sociability (e.g., Warm, α = 0.88), Agency (e.g., Self-reliant, α = 0.71), Animacy (e.g., Alive, α = 0.70), and Disturbing (e.g., Creepy, α = 0.84). Conversely to Experiment 1, participants rated whether they agreed or disagreed (scale from 1 to 7) with a given characteristic assigned to a robot selected randomly between a NAO and a Pepper (the robot was pictured above the scale). The purpose was to test whether the tendency of participants to adopt the intentional stance towards iCub, correlates with the likelihood to attribute anthropomorphic characteristics to robots in general.

### Results

#### Confirmatory Factor Analysis

**FIGURE 5:**
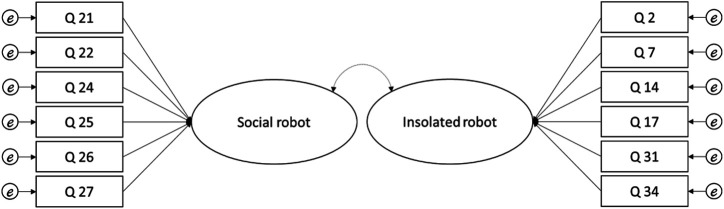
Structural model.

To test the validity of the structure proposed in Experiment 1, we conducted a confirmatory factor analysis (CFA) with a structural model using AMOS plugin in SPSS (Figure 5) (Kline, 2015; Loehlin ad Beaujean, 2016; Wood, 2008) using a variance-covariance matrix with maximum likelihood (ML) estimation (Mishra, 2016). ML estimation is more reliable in many cases than others and is widely used (Bollen, 1989). The model-fit indices showed that chi square (χ2) value was 62.08 (df = 51, *p* = 0.161). Table 6 shows the model-fit indices (Schermelleh-Engel et al., 2003; Jackson et al., 2009) as well as the recommended thresholds (Wood, 2008). As a comparison, we also provide the CFA on one single factor. Although the indices seem similar, they still fit better to the two-factors model compared to a single-factor model.

**TABLE 6:** Confirmatory model fit indices. χ^2^/df the ratio of chi square to degree of freedom; NFI the normalized fit index, CFI the comparative fit index; Tucker–Lewis index (TLI); root mean square error of approximation (RMSEA); **S**RMSR the standardized root mean square residual. Values that do not reach their recommended threshold appear in italic.

	Recommended value	Two factors	Single factors
Chi2/df	≤3.00	1.44	1.56
NFI	≥0.90	0.80	0.77
CFI	≥0.90	0.92	0.90
TLI	≥0.90	0.90	0.87
RMSEA	≤0.08	0.06	0.06
90%CI		[0.02, 0.08]	[0.04, 0.09]
SRMR	≤0.09	0.06	0.07

As shown in Table 6, all model-fit indices exceeded their respective common acceptance threshold except for NFI. NFI represents the goodness of fit through a comparison of the model of interest to a model of completely uncorrelated variables. Still, the value provides evidence in favour of a tested model as good as the saturated model (compared to the independence model) (Bentler and Bonett, 1980). Table 7 presents the non-standardized estimates for each item. All items were significantly associated with their respective factor (all *p*_s_ < 0.001).

**TABLE 7:** CFA standardized estimates.

Items		Factor	Estimate	*p* value
Q2	←	Isolated robot factor	0.44	<0.001
Q7	←	Isolated robot factor	0.48	<0.001
Q14	←	Isolated robot factor	0.41	<0.001
Q17	←	Isolated robot factor	0.46	<0.001
Q31	←	Isolated robot factor	0.34	<0.001
Q34	←	Isolated robot factor	0.40	<0.001
Q21	←	Social robot factor	0.54	<0.001
Q22	←	Social robot factor	0.53	<0.001
Q24	←	Social robot factor	0.65	<0.001
Q25	←	Social robot factor	0.65	<0.001
Q26	←	Social robot factor	0.62	<0.001
Q27	←	Social robot factor	0.65	<0.001

The two factors remained correlated, *r* = 0.54, *p* < 0.001, without reaching the 0.80 threshold at which we cannot differentiate one factor from another (Wood, 2008), arguing that they both efficiently measure the same concept but on separated dimensions or facets. The mean of the social robot factor was 49.19 with a SD = 24.04. The mean of the isolated robot factor was 28.35 with a SD = 17.96. The min-max of the participants’ responses encompasses the 100 points of the slider. Participants’ degree of adopting the intentional stance towards the robot was higher in the social (compared to isolated) robot factor, *t*(134) = 11.66, *p* < 0.001.

#### External Validity

First, to further test the external validity of the IST-2, we conducted a correlation analysis between the IST-2 scores and the negative attitude toward robots (NARS). Results showed significant correlations in the expected direction on both factors for all NARS dimensions (except for the emotional attitudes on factor 2). See Table 8.

**TABLE 8:** Correlation between the present scale factors and NARS dimensions.

		Social/future implications	Emotional attitudes	Actual interactions
Isolated robot factor	Pearson’s r	−0.32	−0.20	0.33
	*p* value	<0.001	0.021	<0.001
Social robot factor	Pearson’s r	−0.24	−0.11	0.36
	*p* value	0.006	0.196	<0.001

To test whether the tendency to adopt the intentional stance, as measured by IST-2, generalizes to the tendency to anthropomorphize other robots, we correlated the IST-2 scores with the anthropomorphic attributions. We processed Pearson correlation analyses including HRIES dimensions (Disturbance, Agency, Sociability and Animacy factors) and the factors of IST-2. Analyses showed a relative predictive power of the social robot factor regarding positive attributions (Agency, Sociability, Animacy) while the isolated robot factor was only significant (with a low Pearson’s *r*) on sociability attributions. Results are presented in Table 9.

**TABLE 9:** Correlation matrix between the two factors and the HRIES factors.

			Disturbing	Agency	Sociability	Animacy
Correlation	Isolated robot factor	Pearson’s r	−0.14	0.08	0.29	0.14
		*p* value	0.117	0.135	0.001	0.107
	Social robot factor	Pearson’s r	−0.05	0.22	0.32	0.21
		*p* value	0.580	0.011	<0.001	0.016
Partial correlation	Isolated robot factor	Pearson’s r	−0.13	-0.11	0.11	−0.008
		*p* value	0.124	0.198	0.217	0.924
	Social robot factor	Pearson’s r	−0.03	0.24	0.22	0.18
		*p* value	0.710	0.005	0.011	0.038

Table 10 presents the correlation matrix for the HRIES dimensions.

**TABLE 10:** Correlation matrix between the four HRIES dimensions.

		Disturbing	Agency	Sociability
Agency	Pearson’s *r*	−0.14	X	X
	*p* value	0.098		
Sociability	Pearson’s *r*	−0.39	0.34	X
	*p* value	<0.001	<0.001	
Animacy	Pearson’s *r*	−0.27	0.43	0.59
	*p* value	0.002	<0.001	<0.001

### Discussion Experiment 2

In Experiment 2 we aimed, first, to confirm the factorial structure found in IST in the first experiment. Second, we tested the external validity of the two factors of IST-2, namely the isolated robot and social robot factors. External validity was tested against a tool to measure general attitudes towards robots. Finally, we investigated the extent to which the tendency to adopt the intentional stance, as measured by IST-2 could predict anthropomorphic attributions towards other robots.

The confirmatory factor analyses assessed the two-factors structure with good-fitting indices. We compared the two-factors structure to a single-factor structure which demonstrated a slightly lower fit. In addition, we found a significant difference in tendency to adopt the intentional stance between the two facets. The reason is, as mentioned earlier, the presence vs. absence of human-robot interaction in the scenarios, which supports the relevance of the two-factors structure.

As predicted, general attitudes were related to the tendency of participants to adopt the intentional stance. The more negative the attitudes, the lower the tendency.

Interestingly, the tendency to adopt the intentional stance (on the social robot factor mainly) was found to predict anthropomorphic attributions towards other robots (Pepper or NAO). However, in comparison to Experiment 1, the correlation indices between the intentional stance tendency and HRIES scores were lower. It is likely that this is because of presenting participants with different robots across the two tasks. However, it remains to be noted that the intentional stance measure in the social robot factor is a better general predictor than the measure in the isolated robot factor. We speculate that this difference might be due to presentation of an interaction in the social scenarios. It might be that the higher focus on the interaction in the social robot factor may result in higher tendency to adopt the intentional stance towards the robot. To test this hypothesis we conducted a post-hoc analysis using the difference between the scores on the social vs. the isolated robot factors as predictor of the dimensions of the HRIES. The results showed that the higher the difference, the higher the agency attributions (r = 0.23, *p* = 0.007).

## General Discussion

People’s attitudes towards robots depend on the extent to which they adopt the intentional stance towards the robots. Attributing mental capacities to an agent, such as goals or intentions, presupposes a (proto-) mind attribution. Past research has demonstrated how the attribution of mind to non-human agents directly influences how individuals consider, behave and accept robots (for reviews see (Epley et al., 2007; Urquiza-Haas and Kotrschal, 2015; Spatola, 2019a; Perez-Osorio and Wykowska, 2020). Therefore, with respect to the increasing presence of robots in human environments, it is crucial to measure, and better understand the likelihood that an individuals will attribute a mind to robots.

We conducted two experiments to validate a task (the Intentional Stance task, IST) that measures a tendency to adopt the intentional stance, without relying on direct explicit questions regarding the construct of interest (Bartneck et al., 2009; Carpinella et al., 2017; Spatola et al., 2020). In other words, IST does not rely on a direct question “to what extent is the robot an intentional agent”, but on a comparison process between two descriptions of robot actions, with one description using mentalistic vocabulary and the other mechanistic terms. Based on items designed by Marchesi et al. (2019) we developed a task (IST-2) encompassing two factors 1) the robot acting in isolation (isolated robot) and 2) the robot interacting with human characters (social robot). Interestingly, the “isolated” behaviours elicit smaller likelihood of adoption of the intentional stance than the “social” ones. This result echoes the “complexity approach” in mentalization theory (Dennett, 1971; Epley et al., 2007) in which the higher the complexity, the higher the likelihood to mentalize the action of an agent as a strategy to reduce complexity (and related uncertainty). Interactions are a more complex phenomenon to represent and understand than isolated behaviours and therefore 1) require more processing and 2) activate social cognition mechanisms (Oberman et al., 2007; Rosset, 2008; Jack et al., 2013). A strategy to deal with this higher degree of complexity is to adopt an intentional stance which allows for a simple and familiar way of making sense of the observed scene (Dennett, 1971). Observing an interaction also activates the social cognition system that (compared to its physical cognition system counterpart) deals with mental properties and intentional goals of actions rather than mechanistic descriptions (Jack et al., 2013).

The two-actors structure of IST-2 raises an important question whether the isolated and social factors are two separate dimensions or rather two facets of a common construct. Considering the factors as separate dimensions would entail that the two factors could be decorrelated. On the contrary, while considering them as two facets of a common construct would presuppose that they are intrinsically related. Partial correlations^7^ argue that each factor can be considered as a separate dimension. However, our results cannot be conclusive regarding this point at this point and further investigation are needed.

The results of both experiments also showed that the tendency to adopt the intentional stance correlated with the anthropomorphic attributions of agency when these agency attributions were related to the same robot (Experiment 1) and to other robots (Experiment 2). These results confirm that intentional stance and anthropomorphism rely on similar constructs (e.g., social cognition) but remain distinct based on the low-medium correlation indices. Interestingly, the field of HRI actually lacks detailed definition and comparison between these concepts (i.e. intentional stance and anthropomorphism) and we argue that providing a framework of such general phenomena, could help to better understand and measure human cognition and behaviour in HRI.

### Limitation

The present study has some limitations that need to be addressed in future research. First, the use of mechanistic and mentalistic sentences in a bidimensional scale assumes a direct opposition between the two concepts on a continuum. On the one side of the continuum, mechanistic reasoning would rely on the physical cognition system while, on the other side, mentalistic reasoning would depend on the social cognition system (Mars et al., 2012; Jack et al., 2013). However, we acknowledge that this is only an assumption and that intentional and mechanistic reasoning might not be in opposition, might not be exclusive, and the transition from one to the other might be gradual and fluid. To what extent these two attitudes are in opposition remains to be answered in future research.

Another limitation of the present study is the lack of divergent/discriminant validity test. In the present experiments, we provided correlation to three measures (HRIES, need for cognition and need for closure, NARS). While the HRIES scale measures attribution of agency (e.g. intention) to a robot, it could be interesting to delineate which specific cognitive constructs are related to the choices of participants in the task. For instance, Kozak and colleagues proposed a framework for mind attribution encompassing 1) the capacity for emotion (e.g., ability to feel pleasure, sadness), 2) attribution of intention (e.g., goals, planning), and 3) the higher-order cognition (e.g., thought, memory) as the process by which an organism acquires awareness of events and objects in its environment (Kozak et al., 2006). Future studies should investigate the relation between IST-2 and the attributions of mind capacities.

Furthermore, the content of the (mechanistic and mentalistic) sentences, while sharing a common dimension, as shown by the internal validity analyses, are more complex than an adjective and therefore are semantically less stable. For instance, the sentence “iCub was unbalanced for a moment/iCub was trying to cheat by looking at opponent’s cards”, triggers more than a mechanistic vs. mentalistic reasoning. Indeed, the fact that the robot is cheating may be understand as a “will” and the thus as a “mentalistic” action. However, it can also be morally valued (cheating is considered as morally negative value). Morally valued behaviour, in turn, s may have an effect on how much of “social”/anthropomorphic traits are attributed to the agent (Spatola et al., 2018).

Finally, the tendency to adopt the intentional stance was analysed in relation to anthropomorphism toward other robots. However, all robots shared a similar “human-like shape” and level of human-likeness (Phillips et al., 2018). Future research might aim at correlating the tendency to adopt the intentional stance towards robots across the entire all human-likeness continuum, in order to argue for a generalization of the “intentional” tendency.

## Conclusion

Understanding the degree to which intentional stance is adopted towards robots is crucial if we would like to better understand the inter-individual differences in engagement in HRI and in acceptance of robots in situations such as schools or workplaces. By providing a tool to measure tendency to adopt the intentional stance, we aim to contribute to this crucial trend in HRI research.

## Data Availability

The datasets presented in this study can be found in online repositories. The names of the repository/repositories and accession number(s) can be found below: https://osf.io/z2kpc/.

## Footnotes

1 Exploratory Factor Analysis (EFA) is a technique that allows the latent structure of a mass of data to be revealed. The latent structure represents a number of factors that explain why some of the variables included in the analysis are cross-correlated, while other variables are not. The latent factors are not directly observable, but the experimenters infer them by taking into account the correlation pattern observed between the variables.

2 The items were translated following a back-translation procedure with two independent translators.

3 To ensure that the sentences were actually characterized as mechanistic or mentalistic, Marchesi and colleagues required 14 volunteers who had a degree in philosophy to rate on a bidimensional 10-point Likert scale how much they understood each sentence as mechanistic vs mentalistic, where 10 denotes completely mentalistic and 0 completely mechanistic. The average score for the mentalistic sentences was 8.2 and 4.3 for the mechanistic sentences (Marchesi et al., 2019).

4 Using orthogonal rotation (e.g. VARIMAX), we preserve the independence of the factors. With oblique rotation (e.g. OBLIMIN, PROMAX), we break it and factors are allowed to correlate.

5 This value is appropriate for most analysis (Hendrickson and White, 1964).

6 The Two-Step cluster analysis is a hybrid approach which first uses a distance measure to separate groups and then a probabilistic approach to choose the optimal subgroup model. The main advantage of this technique is the determining the number of clusters based on a statistical measure of fit (AIC or BIC) rather than on an arbitrary choice (Kent et al., 2014).

7 In Experiment 1, correlation of each factor with HRIES remains significant while controlling for the other factor. In Experiment 2, the social robot factor remains significant, while controlling for the isolated robot factor
